# Modelled impact of a multi-cancer early detection screening programme on cancer treatment in England

**DOI:** 10.1038/s41416-026-03412-2

**Published:** 2026-04-24

**Authors:** Libby Ellis, Charlotte Eversfield, Ewan Gray, Peter S. Hall, Sara Hiom, David A. Jones, Lennard YW Lee, Sean McPhail, Katie Spencer, Christopher Halloran

**Affiliations:** 1 GRAIL Bio UK Ltd, London, UK; 2 National Disease Registration Service, NHS England, London, UK; 3 University of Edinburgh, Edinburgh, UK; 4 University of Oxford, Oxford, UK; 5 University of Liverpool, Liverpool, UK

**Keywords:** Cancer epidemiology, Cancer screening

## Abstract

**Background:**

Cancer screening can reduce late-stage diagnoses, expand treatment options, and improve cancer outcomes. We modelled how introducing a multi-cancer early detection (MCED) screening programme in England could impact cancer treatment patterns.

**Methods:**

The proportions of cancers (19 types, diagnosed 2014–2019) treated with resection, radiotherapy, and systemic anti-cancer therapy (SACT) were applied to modelled stage-specific cancer incidence data with and without addition of MCED screening to existing screening. We modelled an initial screening round (first screen for individuals aged 50–79 years) and a steady-state programme (annual screening from age 50–79 years).

**Results:**

Assuming test parameters are accurate, if MCED screening is introduced in England, more cancers would require resection compared with current annual usage (steady-state: +8900, +10.0%). The number of cancers receiving radiotherapy would decrease overall (–1200; –2.0%) due to a decrease in palliative radiotherapy (–2100; –23.0%); the number of cancers treated with curative radiotherapy would increase slightly (+932; +2.1%). Fewer cancers would receive cytotoxic chemotherapy (–5300, –9.8%) and non-cytotoxic SACT (–530, –12.2%). Increased use of curative treatment combinations is also predicted.

**Conclusions:**

Changes to future service delivery and workforce planning will be needed for the full benefits of an MCED screening programme to be realised.

## Background

Cancer mortality could be substantially reduced if fewer cancers were diagnosed at late stages (usually stage III or IV), when prognosis, treatment options, quality of life and survival are generally worse than for cancers diagnosed at earlier stages [1–3]. However, the proportion of cancers diagnosed at a late stage in England has remained at approximately 46% since 2013 [4]. Thus, substantial innovation is required to change the status quo.

Multi-cancer early detection (MCED) tests screen simultaneously for two or more cancer types using a biological specimen (e.g. blood) to detect cancer signals before symptomatic presentation and diagnosis in usual care [5–7]. Some can also predict the tissue type or organ associated with the cancer signal [5]. The clinical utility of one blood-based MCED test (Galleri^®^; GRAIL, Inc., Menlo Park, CA, USA) for population screening in asymptomatic individuals aged 50–79 years in England is being assessed in the large, randomised controlled NHS-Galleri trial (NCT05611632) [5].

Modelling has predicted that in England, adding annual MCED screening to existing single-cancer screening programmes has the potential to reduce late-stage (stage III and IV) cancer incidence and cancer mortality by 201 and 74 per 100,000 persons, respectively [8]. However, realising these potential mortality benefits depends on people receiving optimal and timely treatment following a cancer diagnosis. Treatment options depend on cancer type and stage, and patient characteristics (e.g. age, comorbidity), and may include various combinations of surgery, radiotherapy, chemotherapy and targeted therapies [9]. The recommendation and use of novel treatments is increasing rapidly, especially for lung, breast and colorectal cancers [10]. increasing the potential for better outcomes for patients. However, there is currently little evidence on how MCED screening could impact cancer treatment.

This study aimed to estimate how the cancer treatment landscape might change if implementation of an MCED screening programme in England reduced late-stage incidence in line with modelled predictions. Realising the full benefits of earlier cancer detection will require careful prioritisation of finite healthcare resources. Our findings will inform discussions about how service delivery and workforce planning might need to be adapted to anticipate potential future demand if an MCED screening programme is offered alongside existing screening programmes.

## Methods

### Data

**Fig. 1:**
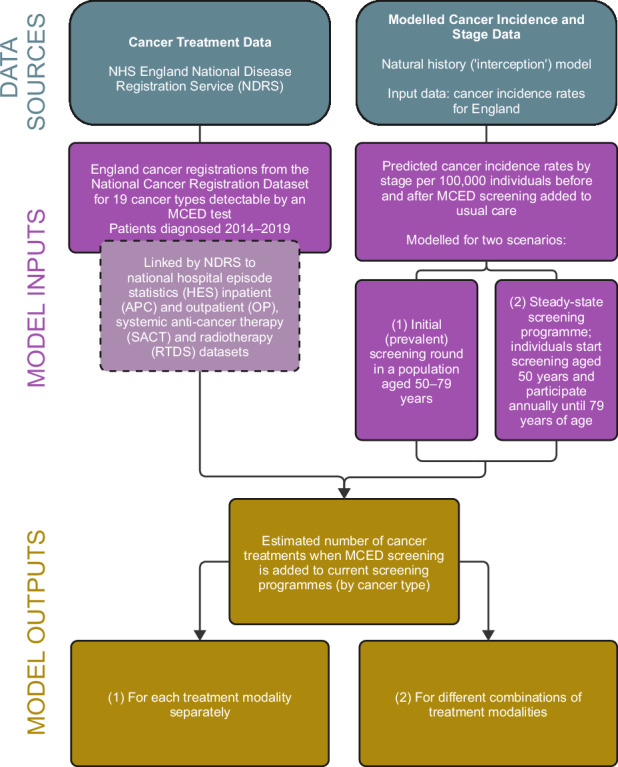
Overview of methods used in this study.

As model inputs, this study used two sets of population-based, routinely collected health data for England (Fig. 1).

The first comprised cancer treatment data by stage for 19 common cancer types detectable by the Galleri MCED test, diagnosed between 2014 and 2019 (Supplementary Table S1). This 5-year period was chosen to minimise any impact of the COVID-19 pandemic on cancer services. Treatment flags for incident cases were created by the National Disease Registration Service (NDRS), using the Cancer Analysis System Standard Operating Procedure (CAS-SOP 4.9) [11]. Treatment flags for tumour resection, radiotherapy, and systemic anti-cancer therapy (SACT), hereafter collectively termed ‘treatment modalities’, in the 18 months following diagnosis were created via linkage to tumour-level (for patients with multiple cancers diagnosed less than 18 months apart) and patient-level treatment tables from the national Radiotherapy Data Set (RTDS), SACT dataset, and inpatient and outpatient hospital episode statistics (HES). Adjuvant and neo-adjuvant treatments were captured, as well as the primary treatment, and tumours which received the same treatment more than once were only counted once. Detailed information on radiotherapy episode (dose and fraction), or number of separate SACT regimens for each tumour, was not available. These data were collected by the NHS as part of the care and support of patients, and collated, maintained and quality-assured by NDRS.

Treatment modality categories were not mutually exclusive (i.e. cancers could be included in more than one category). For each tumour treated with SACT, treatment was categorised as cytotoxic if the earliest listed treatment regimen in the SACT dataset contained any mention of cytotoxic drugs. All other SACT treatments were classified as ‘non-cytotoxic SACT’, including immunotherapy and targeted therapies. If a patient received a combination of therapies in a regimen that included cytotoxic SACT, such as cytotoxic and biological therapies, the patient was categorised as having received cytotoxic SACT. Treatment intent (curative or palliative) in the RTDS and SACT dataset was determined by the intent listed for the earliest SACT regimen or RTDS appointment for each patient. Treatment intent could not be determined for patients with more than one tumour recorded in the 18-month period, because these data could not be linked at the tumour level. A small number of patients had multiple treatment intents recorded as part of the earliest regimen, in which case intent was taken to be palliative.

The second dataset comprised cancer incidence by stage for England for 2014–2019 (Fig. 1). A previously-published natural history (‘interception’) model [8, 12] was used to estimate the change in cancer incidence by stage after MCED screening for two scenarios (Supplementary Table S2). The first was an initial (prevalent) screening round, representing a population aged between 50 and 79 years attending their first MCED screen. This age range was selected to encompass typical cancer screening eligibility, and to align with the NHS-Galleri trial. The second was a steady-state screening programme, representing a population in which individuals enter an MCED screening programme at 50 years of age and participate in annual screening until 79 years of age. In the second scenario, age-specific incidence rates [8]. were applied at each screening round, and the proportion of MCED-detectable cancers detected (‘intercepted’) at each cancer stage by MCED screening was predicted based on an annual screening schedule. The interception model used cancer type- and stage-specific sensitivity estimates specific to the Galleri MCED test to calculate the proportion of MCED detectable cancers at prior stages [13]. Dwell time estimates were cancer-specific [12] with cancers spending 1–2 years in stage I. MCED screening participation was assumed to be 70%, to reflect approximate participation rates in existing standard-of-care screening programmes in the NHS in England [14]. A 100% participation scenario was also considered to demonstrate the maximum potential change in treatments if MCED screening were offered alongside existing screening programmes.

### Modelling assumptions

Firstly, the current overall volume of treatments was examined by modality; i.e. the total number of cancers treated with resection, radiotherapy (overall, and separately for curative and palliative intent), and cytotoxic chemotherapy and non-cytotoxic SACT (including immunotherapy and targeted therapies). The stage-shifted cancer incidence data from the interception model were applied to the proportions of cancers treated with each modality, in both the ‘initial screening round’ and ‘steady-state screening programme’ scenarios. This gave the estimated number of cancers treated with each modality if MCED screening was offered alongside existing screening programmes. Absolute and relative changes are reported.

For each scenario, treatment modalities were considered separately, regardless of whether they were used alone or in combination with any other modality, in order to assess the total impact on each modality. Secondly, two distinct (mutually exclusive) combinations of treatment modalities with curative intent (i.e. excluding treatments with palliative intent) were considered: (1) resection with or without other curative therapy (radiotherapy and/or SACT); and (2) curative radiotherapy and/or curative SACT (without resection). Cancers detected via MCED screening were assumed to have received the same treatments by cancer type and stage as those diagnosed in current standard-of-care practices.

#### Ethical approval and consent to participate

This study used aggregate, national, routinely-collected data collated by the National Disease Registration Service (NDRS). Ethics approval was therefore not required. NDRS has a special legal instruction to collect patient data without consent under section 254 of the Health and Social Care Act 2012.

## Results

Based on cancer incidence data in England for the period 2014 to 2019, we estimated that approximately 188,200 incident cancers would be diagnosed per year (prior to the introduction of MCED screening). Following the initial introduction of MCED screening, a higher cancer detection rate was predicted (~216,600 per year), returning to expected levels in a steady-state screening programme (~188,800 per year).

### Treatment landscape after introduction of MCED screening for each treatment modality

#### All tumour resection

**Fig. 2:**
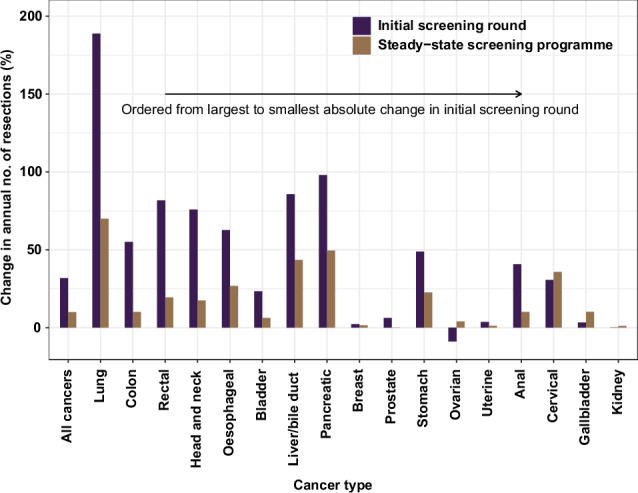
Modelled change in volume of tumour resections in an initial round of multi-cancer early detection (MCED) screening and a steady-state MCED screening programme, when offered alongside current screening programmes in England (assuming 70% participation).

Following the initial introduction of MCED screening, our model predicted approximately 28,000 more tumour resections for all cancer types combined (+31.9%), compared with current annual use (Fig. 2). This was driven by large predicted increases in the number of resections for lung (+10,300; +188.8%), colon (+6600; +55.1%), rectal (+3900; +81.8%), and head and neck (+2900; +75.8%) cancers.

In a steady-state screening programme, our model predicted an attenuated increase, with approximately 8900 more resections required each year for all cancer types combined (+10.0%) compared with current annual usage (Fig. 2), driven by the same four cancer types that drove the initial increase.

Large relative increases in resection in the initial screening round were also predicted for some less common cancers, including pancreatic (+700; +98.0%), liver and bile duct (+720, +85.7%), and oesophageal (+760, +62.7%) cancers (Fig. 2).

#### Radiotherapy

##### Overall

**Fig. 3:**
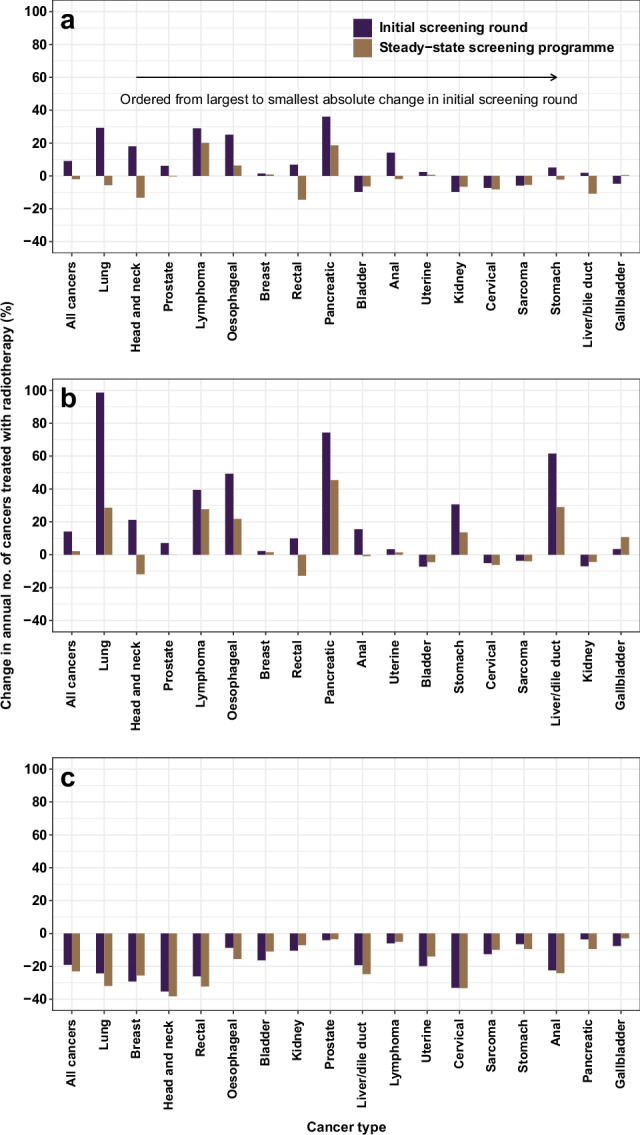
Modelled change in volume of radiotherapy. **a** Overall and with **b** curative and **c** palliative intent in an initial round of multi-cancer early detection (MCED) screening and a steady-state MCED screening programme, when offered alongside current screening programmes in England (assuming 70% participation).

After the initial introduction of MCED screening, there was a predicted increase in the number of cancers treated with radiotherapy (all intents) compared with current annual use (approximately +5500; +9.1%; Fig. 3a). In a steady-state screening programme, the direction of change was reversed, with a small decrease in the number of cancers treated with radiotherapy overall (–1200; –2.0%).

##### Curative

Initially, there was a predicted increase in the number of cancers treated with curative radiotherapy compared with current annual use (approximately +6400; +14.1%; Fig. 3b). There were increases in curative radiotherapy for most cancer types, most notably for lung cancer (+3000; +98.6%), head and neck cancer (+800; +21.2%), and lymphoma (+500; +39.4%). There was also a large absolute increase in curative radiotherapy for prostate cancer, although this represented only a small relative increase (+780; +7.0%).

In a steady-state screening programme, absolute increases in the number of cancers treated with curative radiotherapy were smaller than after the initial screening round (Fig. 3b). For head and neck cancers, the direction of change was reversed, with a decrease in the number of cancers treated with curative radiotherapy (–470; –11.8%).

##### Palliative

Initially, there was a predicted reduction in the number of cancers treated with palliative radiotherapy compared with current annual use (approximately –1700; –19.1%; Fig. 3c). Reductions were predicted for every cancer type, most notably lung cancer (–1100; –24.2%). A similar pattern (–2100; –23.0%) was predicted in a steady-state screening programme, with larger reductions than after the initial screening round.

#### Systemic anti-cancer therapy (SACT)

##### Cytotoxic chemotherapy

**Fig. 4:**
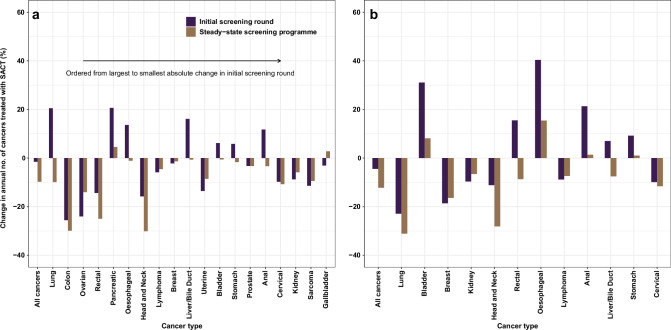
Modelled change in volume of SACT. **a** cytotoxic chemotherapy and **b** non-cytotoxic systemic anti-cancer therapy (SACT) treatments in an initial round of multi-cancer early detection (MCED) screening and a steady-state MCED screening programme, when offered alongside current screening programmes in England (assuming 70% participation).

When MCED screening was initially introduced, there was a small predicted reduction in cytotoxic chemotherapy for all cancers combined compared with current annual use (approximately –830; –1.5%; Fig. 4a). However, the pattern of change varied by cancer type. There were large absolute reductions in the number of cancers treated with cytotoxic chemotherapy for colon (–1700; –25.6%), and, to a lesser extent, ovarian (–660; –24.0%) cancers. In contrast, there were large absolute increases for lung (+1900; +20.5%) and, to a lesser extent, pancreatic (+440; +20.6%) cancers.

In a steady-state screening programme, there was a larger predicted reduction in the number of cancers treated with cytotoxic chemotherapy compared with current annual use (approximately –5300; –9.8%; Fig. 4a). There were reductions for almost all cancer types examined, most notably for colon (–1900; –29.9%), lung (–900; –9.9%), rectal (–800; –25.0%), and head and neck (–670; –30.1%) cancers. There was a notable, albeit small, increase in cytotoxic chemotherapy for pancreatic cancers (+100; +4.5%).

##### Non-cytotoxic SACT

The absolute number of cancers treated with non-cytotoxic SACT in our dataset was small. The predicted reductions were also small compared with current annual use, both after the initial introduction of MCED screening (approximately –200; –4.5%; Fig. 4B) and in a steady-state programme (approximately –540; –12.2%), though patterns varied by cancer type. The only notable change in use was a reduction in the number of lung cancers treated with non-cytotoxic SACT, both in the initial screening round (–220; –22.9%), and in the steady-state screening programme (–300; –31.1%).

### Specific curative treatment combinations after introduction of MCED screening

#### Resection with or without other curative therapy (radiotherapy and/or SACT)

**Fig. 5:**
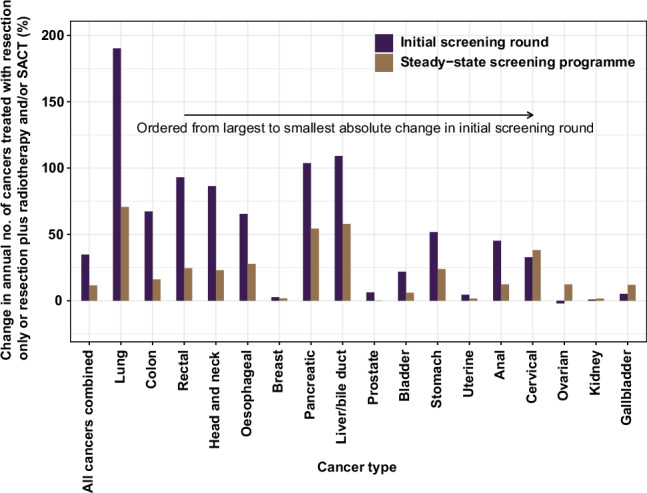
Modelled change in volume of resections with or without other curative radiotherapy and/or systemic anti-cancer therapy (SACT; palliative therapies excluded) in an initial round of multi-cancer early detection (MCED) screening and a steady-state MCED screening programme, when offered alongside current screening programmes in England (assuming 70% participation).

After the initial introduction of MCED screening, the number of cancers treated with resection with or without other curative radiotherapy and/or SACT (i.e. excluding palliative therapies) was predicted to increase by approximately 27,100 (+34.8%) for all cancer types combined, compared with current annual use (Fig. 5). There were increases of varying magnitude for almost all cancer types but most notably for lung (+9400; +190.3%), colon (+6900; +67.3%), rectal (+3800; +93.1%), and head and neck (+2900; +86.4%) cancers. There were also large relative increases in this treatment modality for some less common cancers, in particular for those with a conventionally poor prognosis, including oesophageal (+660; +65.4%), pancreatic (+590; 103.7%), and liver and bile duct (+490; +109.2%) cancers.

In a steady-state screening programme, the predicted increase in the number of resections with or without other curative therapy was attenuated for all cancer types combined (approximately +9000; +11.6%; Fig. 5).

#### Curative radiotherapy or curative chemoradiotherapy (without resection)

**Fig. 6:**
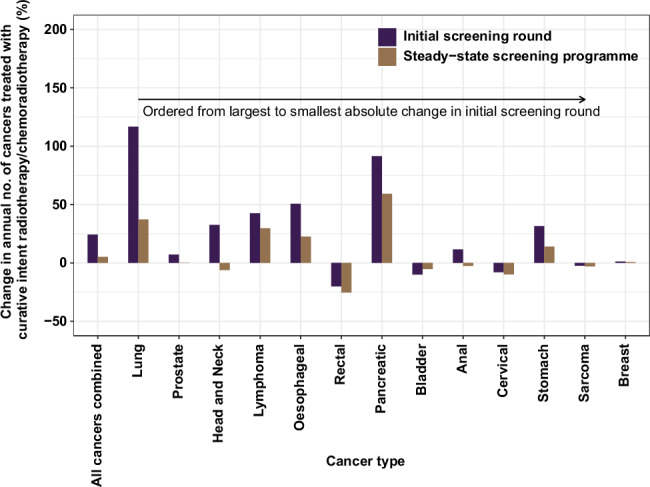
Modelled change in volume of curative radiotherapy or curative chemoradiotherapy treatments without resection, in an initial round of multi-cancer early detection (MCED) screening and a steady-state MCED screening programme, when offered alongside current screening programmes in England (assuming 70% participation.

Initially, there was a predicted increase in the number of cancers treated with curative radiotherapy or curative chemoradiotherapy without resection for all cancer types combined (approximately +4500; +24.3%; Fig. 6). This was mainly driven by large increases for lung cancer (+2500; +116.8%), and smaller increases for head and neck (+610; +32.5%) and prostate cancer (+730; +7.2%), though the relative increase was much smaller for the latter.

In a steady-state screening programme, the predicted increase in the number of cancers treated with curative therapy without resection was attenuated for all cancer types combined (approximately +960; +5.2%; Fig. 6).

Full results tables showing the modelled changes in different treatment modalities and modality combinations when MCED screening was offered alongside existing screening programmes are provided in Tables S2–S17, both for 70% screening participation, to match the data presented in this section, and for 100% screening participation.

## Discussion

Introduction of an MCED screening programme alongside existing single-cancer screening programmes in England would likely reduce the number of cancers diagnosed at a late stage [8, 12]. Our modelling predicts that this reduction in late-stage incidence could result in greater use of curative therapies, i.e. surgical resections, curative radiotherapy, and curative chemoradiotherapy, and reduced use of palliative therapies.

Our study estimated that, given the cancer screening pathways in place during the study period (2014–2019), surgical resections would increase if an MCED screening programme is offered alongside existing screening programmes, in particular for lung, colon, rectal, and head and neck cancers. This is because the Galleri MCED test has relatively high sensitivity for these cancer types, even at early stages [13], which are also relatively common: test sensitivity at stage II is 79.5% for lung, 85.0% for colorectal, and 82.4% for head and neck cancers. In general, early-stage cancers are more treatable, and resectable early-stage cancers have better long-term survival outcomes than resectable late-stage cancers [15, 16]. Thus, in this respect, patient outcomes could potentially improve with the introduction of MCED screening.

In line with the increase in resections, the use of curative radiotherapy would also likely increase if an MCED screening programme is offered alongside existing screening programmes, particularly for lymphoma, and for lung and head and neck cancers, while the use of palliative radiotherapy is likely to decrease. NHS radiotherapy services in England are currently under pressure due to a number of factors, such as workforce shortages and equipment concerns [17]. Further investment in radiotherapy services, including capital investment, will be required to meet the predicted changes in demand. Whilst increases in demand for curative radiotherapy may be partially offset by a decrease in palliative radiotherapy, differences in treatment techniques and fraction numbers between treatment courses with curative and palliative intent mean this is unlikely to be an equal trade-off.

In line with the reduction in late stage presentations, the number of patients with cancer receiving SACT, especially cytotoxic chemotherapy, would likely reduce in both the short and long term if MCED screening is introduced, particularly for colon, rectal, head and neck, and lung cancers. This could not only improve survival but also reduce the detrimental impact of cytotoxic chemotherapy on patients’ quality of life [18]. It is worth noting that based on current patterns of care, the modelled estimates of the impact of MCED screening on SACT use are likely conservativeas only changes in first-line therapy were modelled. Any reduction in late-stage cancer would be expected to reduce the probability of disease progression events following initial treatment, at least in some tumour groups, thus reducing the use of systemic therapy further in the medium to long term.

These long-term changes in SACT use are uncertain, however, given the considerable changes since the time period covered in these analyses (2014–2019). The number of SACT treatments administered each month in England increased between January 2019 and November 2022 for colorectal (17.1%), gynaecological (39.2%), head and neck (38.6%), lung (27.8%), and haematological cancers (37.9%), including lymphoma [19]. Further, evidence for the presence of circulating tumour DNA (ctDNA) as a prognostic indicator is growing [20–25]. Treatment approaches might differ in the future based on whether a cancer is ctDNA^+^ or ctDNA^–^, even for cancers of the same type and stage. For example, ctDNA^+^ cancers may warrant greater use of adjuvant chemotherapy alongside resection [26], potentially offsetting some of the reductions in SACT use predicted in our modelling. On the other hand, ctDNA^+^ cancers are likely to be clinically significant, so the risk of overdiagnosis and overtreatment of indolent cancers through ctDNA-based MCED screening is low.

Beyond this, the wider treatment landscape has changed since the period on which this model is based. Treatment modalities used for lung cancer in particular could change considerably if MCED screening is introduced in the future, with increases in the number of cancers receiving resections and curative radiotherapy and decreases in those receiving SACT. However, the estimated changes might materialise differently in practice, given the advances in lung cancer detection and treatment since our study period. For example, the rollout of targeted lung health checks, which has resulted in an increase in lung cancers diagnosed at an early stage, has been shown to impact downstream treatment practices [27]. The widespread use of curative stereotactic radiotherapy for medically inoperable, early-stage non-small cell lung cancer (NSCLC) [28] also began late on in the study period. The National Institute for Health and Care Excellence (NICE) has also made 37 new treatment recommendations for lung cancer since 2018 [10], including the use of immunotherapy (pembrolizumab for advanced NSCLC, atezolizumab for early or advanced NSCLC) and targeted therapies (e.g. sotorasib for NSCLCs with a *KRAS* mutation). The adoption of new treatments following their approval has also significantly increased over this period; the targeted therapeutic agent osimertinib was previously available only via the Cancer Drugs Fund, but has recently been made routinely and broadly available on the NHS [29].

Treatment advances for other cancer types have also been incorporated into cancer care in England since 2019. Pembrolizumab has been approved for use in metastatic colorectal cancers with high microsatellite instability or mismatch repair deficiency in 2021 [30]. Novel immuno-oncology treatments have transformed the landscape for upper gastrointestinal cancers, including oesophageal cancers: pembrolizumab [31] and nivolumab [32] were both introduced in 2021 to treat late-stage cancers of this type. Polatuzumab [33] and CAR-T cell therapy [34] have both been introduced for non-Hodgkin’s lymphoma, which has improved progression-free survival [35, 36]. It may be that, while the use of cancer treatments primarily indicated for late-stage cancer may decrease following the introduction of an MCED screening programme, there could be an increase in use at earlier stages.

Further, advances in single-cancer screening and early detection, such as lowering the referral threshold at which the faecal immunochemical test (FIT) for colorectal cancer is positive and the roll-out of targeted lung cancer screening, will also impact downstream treatment patterns. We attribute the estimated increase in curative treatment to an increase in earlier stage diagnosis brought about by MCED screening, but in reality it is likely to be a combined effect of all innovation in the field of early detection. In a resource-constrained environment, careful consideration is required of the opportunity costs of novel technologies; cost–effectiveness should be examined through clinical trials and proactive health service planning is then needed if the potential benefits of early detection innovation are to be realised in practice.

Further uncertainty results from the known geographical variation in the use of different treatment modalities and patient demographics influencing treatment decisions [37, 38]. If MCED screening is offered alongside existing screening programmes, the impact of changes in treatment usage on cancer treatment services would likely also vary geographically. Consequently, resource planning in advance, particularly at the local level, is vital to support successful implementation of any future MCED screening programme and should simultaneously seek to address any current unwarranted variation in care. Roll-out of and participation in screening programmes tends to be more gradual than was modelled in our study; increases in treatment volume would therefore also be more gradual, so any service delivery and workforce changes could likely be made incrementally. Uptake of MCED screening may also be differential between population groups, at least in the initial roll-out phase, which could impact short-term treatment patterns. For example, participation in breast and bowel cancer screening in England varies by socioeconomic group with lower uptake rates in people from more socio-economically deprived areas [39–41]. It is not yet known how uptake of MCED screening will compare to existing cancer screening programmes, but previous research shows that the intention to participate in MCED screening is high among adults aged 50–77 years, and does not vary substantially between those in different sociodemographic groups [42]. In any case, participation may be favourable and less differential if conventional barriers to participation can be addressed.

A key strength of our study is that it leveraged comprehensive, routinely collected, population-level cancer treatment data for England, which were recorded using well-defined standard operating procedures. The granularity of these data allowed us to quantify the volume of treatments by modality overall and in combination, including an exploration of modalities with curative and palliative intents.

However, beyond the challenges to interpretation detailed above, there were also some limitations to our study. Firstly, our findings are predicated on the accuracy of the interception model [8]. The precise nature of stage progression, and hence the potential for reduction in the number of late-stage cancers with MCED screening, is also uncertain for some cancer types. For example, ovarian cancer has an unusual stage profile: most cancers of this type are recorded as stage III, but stage III ovarian cancers are notably diverse, with different prognoses and treatment options. Consequently, it is challenging to accurately predict how the stage profile might change with MCED screening or the downstream impact on treatment for ovarian cancer in this model. A large proportion of poor-prognosis cancers are also recorded as having ‘other care’ in routinely-collected health data; this included cases receiving analgesia, other palliative care, or where no treatment was recorded. Due to the heterogeneity of this treatment category, changes in ‘other care’ are difficult to interpret and are not reported in this study. The impact of MCED screening on the management of cancers that might otherwise have been untreated with one of the modalities considered in this study is therefore unknown. The limitation of incomplete or undetailed information is common to the use of routinely collected health data. Indeed, our dataset lacked granular information on the number of different SACT regimens for each patient, and the dose and fraction of radiotherapy episodes, which vary considerably by cancer type and stage. For example, approximately 19,000 lung cancer patients in England (aged 50–79) were treated with SACT in 2019, with over 26,000 regimens. It is therefore likely that our data underestimates total SACT and radiotherapy treatment volume. Patients diagnosed with cancer via a managed screening route may also have different treatment options to those who present symptomatically, but it was not possible to examine treatment differences by route to diagnosis in this study. Due to small numbers in some strata, we were also unable to examine the influence of age (within the 50–79 year range) at a more granular level, though it is possible that for a given stage and cancer type, treatment options may differ for younger and older patients. Our model predictions are conditional on the selected input values and are not inclusive of sampling uncertainty. The uncertainty in test sensitivity for each cancer type and stage is large in comparison to the uncertainty for overall sensitivity. As new data become available, our model will be updated to understand the granular impact on specific cancer types with improved certainty.

## Conclusions

More patients could be eligible for curative treatment if implementation of an MCED screening programme in England reduced late-stage incidence in line with modelled predictions. More cancer patients would likely undergo resection and curative radiotherapy, and fewer would receive systemic anti-cancer therapy, particularly for solid tumours. The need for palliative treatments would likely decrease. However, it is not yet fully known how the approach to treating ctDNA^+^ cancers, those detectable by MCED screening, may differ from ctDNA^–^ cancers in future.

These modelled changes in treatment patterns if MCED screening is introduced could potentially yield better survival for patients with cancer. The NHS-Galleri trial is underway to assess the clinical utility of MCED screening, however, our study suggests that changes will be needed in future service delivery and workforce planning for the full benefits of an MCED screening programme to be realised. Given existing infrastructure and workforce constraints, it is not too soon to consider, at both the local and national level, what resources will be required to maximise the benefits of earlier diagnosis, including for those cancer types without an existing screening programme.

## Supplementary information

Supplementary Material

## Data Availability

The code used to generate the results in this study are available on request from the corresponding author.
