# Supplementary material for: Modelled impact of a multi-cancer early detection screening programme on cancer treatment in England
Source: Br J Cancer. 2026 Apr 24;135(2):265–72. doi: 10.1038/s41416-026-03412-2 (PMC13310839; doi:10.1038/s41416-026-03412-2)
Supplement: Supplementary file 1 — Supplementary Material [file 41416_2026_3412_MOESM1_ESM.docx]

**Supplementary Materials**

Table S1. Distribution of the main treatment modalities (total %) in the 2014-2019 cancer incidence data, by cancer type and stage. Treatment modalities are not mutually exclusive.

| **Cancer Type** | **Treatment distribution (%) by stage and cancer type** | | | | | |
| --- | --- | --- | --- | --- | --- | --- |
|  | **Treatment** | **Stage I** | **Stage II** | **Stage III** | **Stage IV** | **Unknown** |
| Anal | Surgery | 53.2 | 31.6 | 24.8 | 18.3 | 39.1 |
|  | SACT | 59.7 | 80.5 | 86.1 | 66.0 | 50.2 |
|  | Radiotherapy | 65.4 | 88.3 | 92.6 | 59.7 | 57.8 |
| Bladder | Surgery | 96.7 | 31.7 | 52.2 | 25.8 | 29.6 |
|  | SACT | 50.5 | 55.5 | 43.2 | 41.9 | 31.5 |
|  | Radiotherapy | 4.0 | 46.5 | 28.7 | 30.0 | 15.3 |
| Breast | Surgery | 94.6 | 92.4 | 90.0 | 19.8 | 63.0 |
|  | SACT | 14.6 | 44.3 | 73.8 | 56.5 | 29.2 |
|  | Radiotherapy | 71.4 | 70.3 | 80.5 | 34.5 | 40.9 |
| Cervix | Surgery | 80.4 | 13.7 | 10.0 | 5.9 | 16.0 |
|  | SACT | 23.5 | 79.3 | 73.9 | 57.1 | 47.7 |
|  | Radiotherapy | 33.0 | 93.1 | 86.9 | 57.8 | 53.3 |
| Colon | Surgery | 96.4 | 93.4 | 89.2 | 32.5 | 35.0 |
|  | SACT | 2.6 | 23.3 | 70.3 | 57.7 | 17.2 |
|  | Radiotherapy | 0.7 | 1.7 | 5.0 | 6.0 | 2.5 |
| Gallbladder | Surgery | 38.3 | 55.0 | 47.5 | 12.9 | 17.4 |
|  | SACT | 35.4 | 47.6 | 58.8 | 36.8 | 26.4 |
|  | Radiotherapy | 3.1 | 3.7 | 4.7 | 3.9 | 3.0 |
| Head and Neck | Surgery | 71.7 | 53.5 | 48.1 | 43.9 | 42.6 |
|  | SACT | 8.2 | 18.0 | 46.0 | 51.5 | 19.4 |
|  | Radiotherapy | 35.0 | 65.6 | 79.2 | 78.6 | 40.7 |
| Kidney | Surgery | 69.2 | 84.2 | 90.3 | 25.9 | 43.7 |
|  | SACT | 1.1 | 4.6 | 11.8 | 50.7 | 6.9 |
|  | Radiotherapy | 0.8 | 1.8 | 3.1 | 28.2 | 3.1 |
| Liver/Bile Duct | Surgery | 65.9 | 55.6 | 25.4 | 6.6 | 19.0 |
|  | SACT | 29.2 | 38.8 | 39.7 | 35.7 | 24.7 |
|  | Radiotherapy | 4.5 | 4.7 | 5.3 | 9.3 | 3.4 |
| Lung | Surgery | 60.9 | 55.7 | 14.3 | 1.5 | 9.6 |
|  | SACT | 5.4 | 35.9 | 57.7 | 43.3 | 9.9 |
|  | Radiotherapy | 20.4 | 25.7 | 44.6 | 31.2 | 9.3 |
| Lymphoma | Surgery | n/a | n/a | n/a | n/a | n/a |
|  | SACT | 48.8 | 73.5 | 71.7 | 74.1 | 41.0 |
|  | Radiotherapy | 49.5 | 29.1 | 11.0 | 14.1 | 12.8 |
| Melanoma^b^ | Surgery | 98.1 | 96.6 | 97.0 | 58.0 | 67.9 |
|  | SACT | 0.6 | 7.7 | 34.1 | 59.7 | 11.8 |
|  | Radiotherapy | 0.7 | 4.9 | 10.3 | 28.7 | 19.1 |
| Oesophageal | Surgery | 51.9 | 41.1 | 34.6 | 3.7 | 8.5 |
|  | SACT | 32.2 | 70.3 | 73.8 | 55.0 | 29.11 |
|  | Radiotherapy | 26.9 | 46.7 | 41.9 | 24.8 | 23.91 |
| Ovarian | Surgery | 96.8 | 94.0 | 70.0 | 43.9 | 36.9 |
|  | SACT | 48.7 | 81.6 | 86.3 | 77.7 | 39.2 |
|  | Radiotherapy | 1.0 | 3.5 | 1.5 | 2.5 | 1.4 |
| Pancreatic | Surgery | 26.9 | 54.7 | 15.9 | 1.8 | 8.3 |
|  | SACT | 35.8 | 59.0 | 60.0 | 32.8 | 25.5 |
|  | Radiotherapy | 9.1 | 8.9 | 14.5 | 4.2 | 6.8 |
| Prostate | Surgery | 13.6 | 36.3 | 30.1 | 5.5 | 15.1 |
|  | SACT | 1.0 | 1.2 | 3.5 | 36.3 | 4.6 |
|  | Radiotherapy | 28.2 | 37.3 | 56.6 | 35.8 | 24.1 |
| Rectum | Surgery | 98.0 | 82.6 | 76.7 | 22.1 | 30.8 |
|  | SACT | 10.9 | 39.8 | 78.7 | 68.7 | 24.7 |
|  | Radiotherapy | 17.2 | 36.8 | 60.6 | 39.1 | 25.3 |
| Sarcoma | Surgery | n/a | n/a | n/a | n/a | n/a |
|  | SACT | 7.3 | 16.0 | 21.3 | 44.7 | 13.7 |
|  | Radiotherapy | 21.7 | 44.6 | 60.0 | 36.5 | 22.1 |
| Stomach | Surgery | 58.3 | 55.8 | 50.1 | 3.7 | 14.1 |
|  | SACT | 23.3 | 62.2 | 70.0 | 49.5 | 22.8 |
|  | Radiotherapy | 6.0 | 12.6 | 15.4 | 11.5 | 6.9 |
| Uterine | Surgery | 96.6 | 94.9 | 87.4 | 42.0 | 46.4 |
|  | SACT | 6.1 | 22.1 | 67.1 | 65.6 | 10.3 |
|  | Radiotherapy | 27.6 | 78.2 | 62.3 | 19.5 | 18.6 |

Table S2. Stage distribution (%) by cancer type in the 2014-2019 cancer incidence data, and stage-specific incidence rate ratios (IRR) from a natural history (‘interception’) model for an initial (prevalent) round of MCED screening and a steady-state MCED screening programme.

| **Cancer Type** | **Stage distribution (%)** | | | | | **Initial Screening round IRR** | | | | **Steady-State programme IRR** | | | |
| --- | --- | --- | --- | --- | --- | --- | --- | --- | --- | --- | --- | --- | --- |
|  | **Stage I** | **Stage II** | **Stage III** | **Stage IV** | **Unk** | **Stage I** | **Stage II** | **Stage III** | **Stage IV** | **Stage I** | **Stage II** | **Stage III** | **Stage IV** |
| **Anal** | 12.6 | 24.2 | 37.4 | 9.2 | 16.6 | 3 | 1.5 | 0.6 | 0.4 | 1.7 | 1.3 | 0.7 | 0.5 |
| **Bladder** | 41.9 | 23.0 | 8.6 | 13.5 | 12.9 | 1.4 | 0.9 | 1.0 | 0.6 | 1.1 | 0.9 | 1.2 | 0.7 |
| **Breast** | 45.7 | 34.4 | 7.5 | 4.5 | 7.9 | 1.0 | 1.1 | 0.8 | 0.6 | 1.0 | 1.1 | 0.9 | 0.6 |
| **Cervix** | 28.6 | 26.0 | 9.8 | 15.5 | 20.1 | 1.5 | 0.9 | 0.8 | 0.4 | 1.5 | 0.9 | 0.6 | 0.4 |
| **Colon^a^** | 16.0 | 25.7 | 25.6 | 23.9 | 8.8 | 3.6 | 1.6 | 0.6 | 0.4 | 1.9 | 1.2 | 0.6 | 0.5 |
| **Rectal^a^** | 24.3 | 16.5 | 34.0 | 17.6 | 7.5 |  |  |  |  |  |  |  |  |
| **Gallbladder** | 7.1 | 15.5 | 15.4 | 34.5 | 27.5 | 1.0 | 1.2 | 1.0 | 0.8 | 1.0 | 1.3 | 1.2 | 0.8 |
| **Head and Neck** | 19.4 | 11.6 | 12.6 | 44.6 | 11.8 | 3.9 | 2.6 | 1.1 | 0.4 | 2.3 | 1.5 | 0.8 | 0.5 |
| **Kidney** | 41.3 | 7.0 | 17.6 | 19.1 | 15.1 | 1.0 | 1.1 | 0.9 | 0.9 | 1.0 | 1.2 | 0.9 | 0.9 |
| **Liver/Bile Duct** | 8.8 | 9.6 | 8.9 | 26.7 | 46.1 | 4.5 | 1.2 | 1.1 | 0.4 | 3.0 | 1.0 | 0.8 | 0.5 |
| **Lung** | 18.7 | 7.5 | 20.0 | 47.1 | 6.7 | 2.4 | 6.0 | 1.2 | 0.5 | 1.5 | 3.1 | 0.9 | 0.5 |
| **Lymphoma** | 14.6 | 10.7 | 16.4 | 39.9 | 18.5 | 1.9 | 1.6 | 0.9 | 0.5 | 1.6 | 1.6 | 0.9 | 0.6 |
| **Melanoma^b^** | 61.2 | 18.4 | 7.0 | 2.8 | 10.7 | 1.0 | 1.0 | 1.0 | 1.0 | 1.0 | 1.0 | 1.0 | 1.0 |
| **Oesophageal** | 9.0 | 12.2 | 29.7 | 35.3 | 13.7 | 2.4 | 2.6 | 1.0 | 0.5 | 1.5 | 2.0 | 0.9 | 0.6 |
| **Ovarian** | 17.7 | 6.4 | 39.4 | 23.8 | 12.8 | 1.4 | 1.9 | 0.6 | 0.4 | 1.6 | 2.4 | 0.6 | 0.5 |
| **Pancreatic** | 5.8 | 12.1 | 10.7 | 52.1 | 19.3 | 5.5 | 1.8 | 1.1 | 0.5 | 4.8 | 1.1 | 1.0 | 0.6 |
| **Prostate** | 34.2 | 18.3 | 22.4 | 15.6 | 9.5 | 1.1 | 1.0 | 1.2 | 0.9 | 1.0 | 1.0 | 1.0 | 1.0 |
| **Sarcoma** | 13.2 | 10.7 | 11.8 | 9.9 | 54.5 | 1.5 | 1.0 | 0.8 | 0.6 | 1.4 | 1.0 | 0.8 | 0.7 |
| **Stomach** | 9.9 | 12.8 | 19.0 | 39.8 | 18.4 | 2.4 | 1.7 | 1.1 | 0.7 | 1.6 | 1.5 | 1.0 | 0.7 |
| **Uterine** | 70.3 | 6.0 | 10.1 | 6.2 | 7.3 | 1.1 | 1.2 | 0.8 | 0.6 | 1.0 | 1.1 | 0.9 | 0.7 |

^a^The interception model groups colon and rectal cancers together; these values were applied to both colon and rectal cancers individually in this study

Table S3. Age distribution (%) in the 2014-2019 cancer incidence data, by cancer type and stage

| **Cancer Type** | **Age distribution (%) by stage and cancer type** | | | | | |
| --- | --- | --- | --- | --- | --- | --- |
|  | **Age group** | **Stage I** | **Stage II** | **Stage III** | **Stage IV** | **Unknown** |
| Anal | 50-59 | 31.3% | 29.3% | 33.1% | 28.3% | 28.8% |
|  | 60-69 | 36.2% | 37.1% | 39.8% | 38.5% | 38.6% |
|  | 70-79 | 32.5% | 33.6% | 27.1% | 33.2% | 32.7% |
| Bladder | 50-59 | 10.4% | 10.4% | 12.9% | 12.7% | 9.7% |
|  | 60-69 | 32.2% | 30.8% | 31.5% | 32.4% | 28.3% |
|  | 70-79 | 57.4% | 58.8% | 55.5% | 54.9% | 62.0% |
| Breast | 50-59 | 33.1% | 34.8% | 36.0% | 27.0% | 33.1% |
|  | 60-69 | 41.5% | 34.2% | 32.1% | 31.2% | 31.7% |
|  | 70-79 | 25.3% | 30.9% | 31.9% | 41.7% | 35.2% |
| Cervix | 50-59 | 59.7% | 39.6% | 41.3% | 33.4% | 38.0% |
|  | 60-69 | 28.3% | 33.6% | 30.5% | 33.7% | 32.4% |
|  | 70-79 | 12.0% | 26.8% | 28.2% | 32.9% | 29.6% |
| Colon | 50-59 | 13.4% | 14.6% | 17.7% | 19.5% | 14.1% |
|  | 60-69 | 38.9% | 33.2% | 34.9% | 34.0% | 29.1% |
|  | 70-79 | 47.7% | 52.2% | 47.4% | 46.4% | 56.7% |
| Gallbladder | 50-59 | 14.1% | 17.0% | 16.3% | 14.1% | 13.0% |
|  | 60-69 | 36.4% | 34.9% | 37.0% | 34.1% | 28.2% |
|  | 70-79 | 49.5% | 48.1% | 46.7% | 51.8% | 58.7% |
| Head and Neck | 50-59 | 29.1% | 28.7% | 33.3% | 33.0% | 26.4% |
|  | 60-69 | 38.1% | 38.8% | 39.5% | 40.4% | 38.9% |
|  | 70-79 | 32.9% | 32.5% | 27.2% | 26.6% | 34.7% |
| Kidney | 50-59 | 25.2% | 27.2% | 23.3% | 22.1% | 21.7% |
|  | 60-69 | 37.0% | 37.3% | 38.0% | 37.5% | 32.7% |
|  | 70-79 | 37.8% | 35.5% | 38.7% | 40.4% | 45.6% |
| Liver/Bile Duct | 50-59 | 22.6% | 19.5% | 15.8% | 16.9% | 16.2% |
|  | 60-69 | 36.8% | 38.6% | 36.2% | 36.2% | 36.0% |
|  | 70-79 | 40.5% | 42.0% | 48.1% | 46.9% | 47.8% |
| Lung | 50-59 | 10.6% | 10.3% | 13.4% | 14.1% | 10.3% |
|  | 60-69 | 34.0% | 35.1% | 36.0% | 36.5% | 31.0% |
|  | 70-79 | 55.4% | 54.6% | 50.6% | 49.5% | 58.7% |
| Lymphoma | 50-59 | 22.8% | 24.7% | 22.7% | 19.4% | 18.5% |
|  | 60-69 | 34.8% | 34.8% | 34.7% | 35.1% | 33.5% |
|  | 70-79 | 42.4% | 40.5% | 42.6% | 45.5% | 48.0% |
| Melanoma^b^ | 50-59 | 30.4% | 19.2% | 27.8% | 22.5% | 24.3% |
|  | 60-69 | 35.7% | 33.0% | 33.6% | 35.0% | 34.5% |
|  | 70-79 | 34.0% | 47.8% | 38.6% | 42.5% | 41.2% |
| Oesophageal | 50-59 | 14.8% | 14.7% | 17.1% | 19.1% | 13.5% |
|  | 60-69 | 38.4% | 35.6% | 38.0% | 38.0% | 34.0% |
|  | 70-79 | 46.8% | 49.7% | 44.9% | 42.9% | 52.5% |
| Ovarian | 50-59 | 39.3% | 30.2% | 21.9% | 20.3% | 21.7% |
|  | 60-69 | 34.6% | 33.8% | 36.3% | 35.3% | 30.1% |
|  | 70-79 | 26.1% | 35.9% | 41.9% | 44.3% | 48.2% |
| Pancreatic | 50-59 | 13.6% | 17.1% | 16.9% | 16.1% | 12.9% |
|  | 60-69 | 31.9% | 35.3% | 34.7% | 35.6% | 31.2% |
|  | 70-79 | 54.5% | 47.6% | 48.4% | 48.3% | 55.9% |
| Prostate | 50-59 | 15.8% | 16.6% | 11.3% | 9.7% | 13.9% |
|  | 60-69 | 41.6% | 42.3% | 40.4% | 35.7% | 34.9% |
|  | 70-79 | 42.6% | 41.1% | 48.2% | 54.6% | 51.2% |
| Rectum | 50-59 | 18.1% | 18.2% | 23.7% | 22.6% | 22.2% |
|  | 60-69 | 39.0% | 36.9% | 37.5% | 36.2% | 33.6% |
|  | 70-79 | 42.9% | 44.9% | 38.8% | 41.2% | 44.2% |
| Sarcoma | 50-59 | 33.1% | 28.4% | 27.5% | 29.9% | 26.8% |
|  | 60-69 | 34.7% | 33.2% | 33.4% | 35.2% | 32.7% |
|  | 70-79 | 32.3% | 38.4% | 39.1% | 35.0% | 40.5% |
| Stomach | 50-59 | 14.1% | 17.4% | 18.4% | 17.7% | 15.1% |
|  | 60-69 | 30.3% | 29.5% | 31.9% | 32.8% | 29.4% |
|  | 70-79 | 55.6% | 53.1% | 49.7% | 49.5% | 55.5% |
| Uterine | 50-59 | 27.5% | 24.2% | 21.6% | 18.4% | 24.6% |
|  | 60-69 | 39.7% | 37.1% | 35.5% | 37.7% | 35.1% |
|  | 70-79 | 32.8% | 38.7% | 42.9% | 43.9% | 40.4% |

**Table S4. Modelled changes in the average number of tumour resections per year by cancer type in an initial round of multi-cancer early detection (MCED) screening and a steady-state MCED screening programme, when offered alongside current screening programmes (assuming 70% participation).** Cancer types are ordered from largest to smallest percentage change in the initial screening round.

|  |  | **Initial Screening Round** | | **Steady-State Screening Programme** | |
| --- | --- | --- | --- | --- | --- |
| **Cancer Type** | **Usual Care (n)** | **Usual Care & MCED (n)** | **Change (n [%])** | **Usual Care & MCED (n)** | **Change (%)** |
| **All Cancers Combined** | 88,915 | 117,237 | 28,322 (31.9) | 97,766 | 8851 (10) |
| **Lung** | 5474 | 15,805 | 10331 (188.8) | 9302 | 3828 (69.9) |
| **Pancreatic** | 715 | 1415 | 700 (98.0) | 1068 | 353 (49.5) |
| **Liver & Bile Duct** | 835 | 1550 | 715 (85.7) | 1199 | 364 (43.5) |
| **Rectal** | 4763 | 8657 | 3894 (81.8) | 5686 | 923 (19.4) |
| **Head and Neck** | 3820 | 6716 | 2896 (75.8) | 4486 | 666 (17.4) |
| **Oesophageal** | 1215 | 1976 | 761 (62.7) | 1540 | 325 (26.8) |
| **Colon** | 11,976 | 18,577 | 6601 (55.1) | 13,185 | 1209 (10.1) |
| **Stomach** | 868 | 1292 | 424 (48.9) | 1065 | 197 (22.7) |
| **Anal** | 294 | 413 | 119 (40.7) | 323 | 29 (10.1) |
| **Cervical** | 260 | 340 | 80 (30.7) | 354 | 94 (35.8) |
| **Bladder** | 3112 | 3839 | 727 (23.3) | 3306 | 194 (6.2) |
| **Prostate** | 7232 | 7683 | 451 (6.2) | 7243 | 11 (0.2) |
| **Uterine** | 5358 | 5556 | 198 (3.7) | 5422 | 64 (1.2) |
| **Gallbladder** | 336 | 347 | 11 (3.3) | 370 | 34 (10.2) |
| **Breast** | 26,917 | 27,547 | 630 (2.3) | 27,324 | 407 (1.5) |
| **Kidney** | 4128 | 4135 | 7 (0.2) | 4176 | 48 (1.2) |
| **Ovarian** | 2548 | 2323 | -225 (-8.8) | 2651 | 103 (4.1) |
| **Lymphoma^a^** | NA | NA | NA | NA | NA |
| **Sarcoma^a^** | NA | NA | NA | NA | NA |

^a^Lymphoma and sarcoma have been excluded from this analysis because resection is not a standard of care treatment for these cancer types.

**Table S5. Modelled changes in the average number of radiotherapy treatments (all intents) per year by cancer type in an initial round of multi-cancer early detection (MCED) screening and a steady-state MCED screening programme, when offered alongside current screening programmes (assuming 70% participation).** Cancer types are ordered from largest to smallest percentage change in the initial screening round.

|  |  | **Initial Screening Round** | | **Steady State Screening Programme** | |
| --- | --- | --- | --- | --- | --- |
| **Cancer type** | **Usual Care (n)** | **Usual Care & MCED (n)** | **Change (n [%])** | **Usual Care & MCED (n)** | **Change (%)** |
| **All cancers combined** | 60,841 | 66,356 | 5515 (9.1) | 59,655 | -1186 (-2.0) |
| **Pancreatic** | 382 | 520 | 138 (36.1) | 453 | 71 (18.6) |
| **Lung** | 8282 | 10,704 | 2422 (29.3) | 7814 | -468 (-5.7) |
| **Lymphoma** | 1814 | 2340 | 526 (29.0) | 2179 | 365 (20.1) |
| **Oesophageal** | 1765 | 2208 | 443 (25.1) | 1876 | 111 (6.3) |
| **Head and Neck** | 4831 | 5700 | 869 (18.0) | 4190 | -641 (-13.3) |
| **Anal** | 733 | 837 | 104 (14.2) | 719 | -14 (-1.9) |
| **Rectal** | 2707 | 2893 | 186 (6.9) | 2315 | -392 (-14.5) |
| **Prostate** | 13,185 | 13,998 | 813 (6.2) | 13,148 | -37 (-0.3) |
| **Stomach** | 359 | 377 | 18 (5.2) | 351 | -8 (-2.2) |
| **Uterine** | 1997 | 2044 | 47 (2.4) | 2010 | 13 (0.7) |
| **Liver/Bile Duct** | 187 | 190 | 3 (1.9) | 167 | -20 (-10.8) |
| **Breast** | 20,767 | 21,073 | 306 (1.5) | 20,940 | 173 (0.8) |
| **Gallbladder** | 44 | 42 | -2 (-4.7) | 45 | 1 (0.4) |
| **Sarcoma** | 543 | 511 | -32 (-5.8) | 514 | -29 (-5.4) |
| **Cervical** | 508 | 472 | -36 (-7.2) | 467 | -41 (-8.2) |
| **Bladder** | 1090 | 984 | -106 (-9.7) | 1021 | -69 (-6.3) |
| **Kidney** | 457 | 413 | -44 (-9.7) | 427 | -30 (-6.6) |
| **Ovarian^a^** | NA | NA | NA | NA | NA |
| **Colon^a^** | NA | NA | NA | NA | NA |

^a^Ovarian and colon cancer have been excluded from this analysis because radiotherapy is not a standard of care treatment for these cancer types.

**Table S6. Modelled changes in the average number of curative radiotherapy treatments per year by cancer type in an initial round of multi-cancer early detection (MCED) screening and a steady-state MCED screening programme, when offered alongside current screening programmes (assuming 70% participation).** Cancer types are ordered from largest to smallest percentage change in the initial screening round.

|  |  | **Initial Screening Round** | | **Steady-State Screening Programme** | |
| --- | --- | --- | --- | --- | --- |
| **Cancer Type** | **Usual Care (n)** | **Usual Care & MCED (n)** | **Change (n [%])** | **Usual Care & MCED (n)** | **Change (%)** |
| **All Cancers Combined** | 45,212 | 51,571 | 6359 (14.1) | 46,144 | 932 (2.1) |
| **Lung** | 3010 | 5979 | 2969 (98.6) | 3870 | 860 (28.6) |
| **Pancreatic** | 179 | 313 | 134 (74.3) | 261 | 82 (45.4) |
| **Liver/Bile Duct** | 20 | 32 | 12 (61.5) | 26 | 6 (28.9) |
| **Oesophageal** | 887 | 1324 | 437 (49.3) | 1080 | 193 (21.8) |
| **Lymphoma** | 1268 | 1768 | 500 (39.4) | 1618 | 350 (27.6) |
| **Stomach** | 90 | 117 | 27 (30.6) | 102 | 12 (13.6) |
| **Head and Neck** | 3956 | 4794 | 838 (21.2) | 3487 | -469 (-11.8) |
| **Anal** | 608 | 703 | 95 (15.5) | 603 | 5 (-0.8) |
| **Rectal** | 2116 | 2326 | 210 (9.9) | 1846 | -270 (-12.8) |
| **Prostate** | 11,034 | 11,812 | 778 (7.0) | 11,031 | 0 (0.0) |
| **Gallbladder** | 11 | 11 | 0 (0) | 12 | 1 (10.7) |
| **Uterine** | 1669 | 1724 | 55 (3.3) | 1692 | 23 (1.4) |
| **Breast** | 18,476 | 18,885 | 409 (2.2) | 18,748 | 272 (1.5) |
| **Sarcoma** | 367 | 354 | -13 (-3.6) | 352 | 15 (-4.0) |
| **Cervical** | 411 | 390 | -21 (-5.1) | 386 | -25 (-6.2) |
| **Kidney** | 40 | 37 | -3 (-7.1) | 38 | -2 (-4.4) |
| **Bladder** | 606 | 562 | -44 (-7.3) | 579 | -27 (-4.5) |
| **Ovarian^a^** | NA | NA | NA | NA | NA |
| **Colon^a^** | NA | NA | NA | NA | NA |

^a^Ovarian and colon cancer have been excluded from this analysis because radiotherapy is not a standard of care treatment for these cancer types.

**Table S7. Modelled changes in the average number of palliative radiotherapy treatments per year by cancer type in an initial round of multi-cancer early detection (MCED) screening and a steady-state MCED screening programme, when offered alongside current screening programmes (assuming 70% participation).** Cancer types are ordered from largest to smallest percentage decrease in the initial screening round.

|  |  | **Initial Screening Round** | | **Steady-State Screening Programme** | |
| --- | --- | --- | --- | --- | --- |
| **Cancer type** | **Usual Care (n)** | **Usual Care & MCED (n)** | **Change (n [%])** | **Usual Care & MCED (n)** | **Change (%)** |
| **All Cancers Combined** | 9172 | 7425 | -1747 (-19.1) | 7058 | -2114 (-23.0) |
| **Head and Neck** | 308 | 200 | -108 (-35.2) | 191 | -117 (-38.2) |
| **Cervical** | 46 | 31 | -15 (-33.0) | 31 | -15 (-33.2) |
| **Breast** | 414 | 293 | -121 (-29.2) | 308 | -106 (-25.5) |
| **Rectal** | 278 | 205 | -73 (-26.0) | 188 | -90 (-32.3) |
| **Lung** | 4361 | 3305 | -1056 (-24.2) | 2967 | -1394 (-32.0) |
| **Anal** | 42 | 33 | -9 (-22.4) | 32 | -10 (-24.2) |
| **Uterine** | 87 | 70 | -17 (-19.9) | 75 | -12 (-14.0) |
| **Liver/Bile Duct** | 113 | 92 | -21 (-19.)3 | 85 | -28 (-24.7) |
| **Bladder** | 314 | 262 | -52 (-16.)3 | 279 | -35 (-10.9) |
| **Sarcoma** | 121 | 106 | -15 (-12.6) | 109 | -12 (-9.9) |
| **Kidney** | 364 | 326 | -38 (-10.4) | 338 | -26 (-7.1) |
| **Oesophageal** | 667 | 608 | -59 (-8.7) | 564 | -103 (-15.5) |
| **Gallbladder** | 28 | 26 | -2 (-7.6) | 27 | -1 (-2.9) |
| **Stomach** | 220 | 206 | -14 (-6.5) | 200 | -20 (-9.4) |
| **Lymphoma** | 342 | 321 | -21 (-5.9) | 324 | -18 (-5.1) |
| **Prostate** | 901 | 864 | -37 (-4.1) | 870 | -31 (-3.4) |
| **Pancreatic** | 158 | 152 | -6 (-3.6) | 143 | -15 (-9.4) |
| **Ovarian^a^** | NA | NA | NA | NA | NA |
| **Colon^a^** | NA | NA | NA | NA | NA |

^a^Ovarian and colon cancer have been excluded from this analysis because radiotherapy is not a standard of care treatment for these cancer types.

**Table S8. Modelled changes in the average number of cytotoxic systemic anti-cancer therapy (SACT) treatments per year by cancer type in an initial round of multi-cancer early detection (MCED) screening and a steady-state MCED screening programme, when offered alongside current screening programmes (assuming 70% participation).** Cancer types are ordered from largest to smallest percentage change in the initial screening round.

|  |  | **Initial Screening Round** | | **Steady-State Screening Programme** | |
| --- | --- | --- | --- | --- | --- |
| **Cancer Type** | **Usual Care (n)** | **Usual Care & MCED (n)** | **Change (n [%])** | **Usual Care & MCED (n)** | **Change (%)** |
| **All Cancers Combined** | 54,317 | 53,484 | -833 (-1.5) | 49,020 | -5297 (-9.8) |
| **Pancreatic** | 2146 | 2588 | 442 (20.6) | 2243 | 97 (4.5) |
| **Lung** | 9069 | 10,929 | 1860 (20.5) | 8172 | -897 (-9.9) |
| **Liver/Bile Duct** | 937 | 1088 | 151 (16.1) | 931 | -6 (-0.6) |
| **Oesophageal** | 3001 | 3411 | 410 (13.6) | 2969 | -32 (-1.1) |
| **Anal** | 606 | 677 | 71 (11.8) | 585 | -21 (-3.4) |
| **Bladder** | 2075 | 2203 | 128 (6.2) | 2063 | -12 (-0.6) |
| **Stomach** | 1497 | 1584 | 87 (5.8) | 1472 | -25 (-1.7) |
| **Breast** | 9432 | 9228 | -204 (-2.2) | 9305 | -127 (-1.3) |
| **Gallbladder** | 469 | 454 | -15 (-3.1) | 482 | 13 (2.8) |
| **Prostate** | 2371 | 2294 | -77 (-3.2) | 2293 | -78 (-3.3) |
| **Lymphoma** | 5530 | 5208 | -322 (-5.8) | 5279 | -251 (-4.6) |
| **Kidney** | 396 | 362 | -34 (-8.8) | 373 | -23 (-5.8) |
| **Cervical** | 398 | 359 | -39 (-9.7) | 355 | -43 (-10.7) |
| **Sarcoma** | 294 | 260 | -34 (-11.4) | 266 | -28 (-9.4) |
| **Uterine** | 1023 | 884 | -139 (-13.6) | 935 | -88 (-8.6) |
| **Rectal** | 3182 | 2724 | -458 (-14.4) | 2387 | -795 (-25.0) |
| **Head and Neck** | 2219 | 1869 | -350 (-15.8) | 1550 | -669 (-30.1) |
| **Ovarian** | 2750 | 2089 | -661 (-24.0) | 2364 | -386 (-14.0) |
| **Colon** | 6458 | 4807 | -1651 (-25.6) | 4529 | -1929 (-29.9) |

**Table S9. Modelled changes in the average number of non-cytotoxic systemic anti-cancer therapy (SACT) treatments per year by cancer type in an initial round of multi-cancer early detection (MCED) screening and a steady-state MCED screening programme, when offered alongside current screening programmes (assuming 70% participation).** Cancer types are ordered from largest to smallest percentage change in the initial screening round.

|  |  | **Initial Screening Round** | | **Steady-State Screening Programme** | |
| --- | --- | --- | --- | --- | --- |
| **Cancer type** | **Usual Care (n)** | **Usual Care & MCED (n)** | **Change (n [%])** | **Usual Care & MCED (n)** | **Change (%)** |
| **All Cancers Combined** | 4405 | 4208 | -197 (-4.5) | 3867 | -538 (-12.2) |
| **Oesophageal** | 76 | 106 | 30 (40.4) | 88 | 12 (15.4) |
| **Bladder** | 398 | 521 | 123 (31.1) | 430 | 32 (8.1) |
| **Anal** | 74 | 90 | 16 (21.3) | 75 | 1 (1.4) |
| **Rectal** | 226 | 261 | 35 (15.5) | 206 | -20 (-8.7) |
| **Colon^a^** | 52 | 57 | 5 (10.4) | 46 | -6 (-11.3) |
| **Stomach** | 58 | 63 | 5 (9.2) | 59 | 1 (1.0) |
| **Liver/Bile Duct** | 135 | 145 | 10 (7.0) | 125 | -10 (-7.5) |
| **Pancreatic^a^** | 18 | 18 | 0 (0.0) | 17 | -1 (-4.0) |
| **Prostate^a^** | 271 | 275 | 4 (1.6) | 266 | -5 (-1.8) |
| **Uterine^a^** | 18 | 18 | 0 (0.0) | 18 | 0 (0.0) |
| **Gallbladder^a^** | 1 | 1 | 0 (0.0) | 1 | 0 (0.0) |
| **Sarcoma^a^** | 12 | 11 | -1 (-7.5) | 11 | -1 (-6.2) |
| **Lymphoma** | 226 | 206 | -20 (-8.8) | 210 | -16 (-7.4) |
| **Kidney** | 510 | 461 | -49 (-9.6) | 477 | -33 (-6.6) |
| **Cervical** | 38 | 34 | -4 (-9.9) | 34 | -4 (-11.6) |
| **Head and Neck** | 394 | 350 | -44 (-11.1) | 283 | -111 (-28.1) |
| **Breast** | 473 | 385 | -88 (-18.6) | 396 | -77 (-16.4) |
| **Lung** | 954 | 735 | -219 (-22.9) | 657 | -297 (-31.1) |
| **Ovarian^a^** | 10 | 7 | -3 (-25.9) | 8 | -2 (-17.3) |

^a^Data for these cancer types have been excluded from the figures presented in the main text, because absolute change was negligible.

**Table S10. Modelled changes in the average number of tumour resections with or without other curative therapy (radiotherapy and/or systemic anti-cancer therapy) per year by cancer type in an initial round of multi-cancer early detection (MCED) screening and a steady-state MCED screening programme, when offered alongside current screening programmes (assuming 70% participation).** Cancer types are ordered from largest to smallest percentage change in the initial screening round.

|  |  | **Initial Screening Round** | | **Steady-State Screening Programme** | |
| --- | --- | --- | --- | --- | --- |
| **Cancer type** | **Usual Care (n)** | **Usual Care & MCED (n)** | **Change (n [%])** | **Usual Care & MCED (n)** | **Change (%)** |
| **All Cancers Combined** | 77,752 | 104,818 | 27,066 (34.8) | 86,766 | 9014 (11.6) |
| **Lung** | 4950 | 14,368 | 9418 (190.3) | 8447 | 3497 (70.7) |
| **Liver/Bile Duct** | 445 | 930 | 485 (109.2) | 702 | 257 (57.9) |
| **Pancreatic** | 570 | 1160 | 590 (103.7) | 879 | 309 (54.4) |
| **Rectal** | 4063 | 7847 | 3784 (93.1) | 5059 | 996 (24.5) |
| **Head and Neck** | 3373 | 6287 | 2914 (86.4) | 4147 | 774 (23.0) |
| **Colon** | 10,304 | 17,235 | 6931 (67.3) | 11,968 | 1664 (16.1) |
| **Oesophageal** | 1006 | 1663 | 657 (65.4) | 1285 | 279 (27.7) |
| **Stomach** | 734 | 1114 | 380 (51.8) | 909 | 175 (23.9) |
| **Anal** | 244 | 355 | 111 (45.2) | 274 | 30 (12.4) |
| **Cervical** | 236 | 313 | 77 (32.8) | 325 | 89 (38.2) |
| **Bladder** | 1830 | 2230 | 400 (21.9) | 1942 | 112 (6.1) |
| **Prostate** | 7120 | 7567 | 447 (6.3) | 7133 | 13 (0.2) |
| **Gallbladder** | 252 | 265 | 13 (5.1) | 282 | 30 (11.9) |
| **Uterine** | 4850 | 5074 | 224 (4.6) | 4934 | 84 (1.7) |
| **Breast** | 23,691 | 24,327 | 636 (2.7) | 24,107 | 412 (1.8) |
| **Kidney** | 3772 | 3806 | 34 (0.9) | 3838 | 66 (1.7) |
| **Ovarian** | 1799 | 1764 | -35 (-2.0) | 2020 | 221 (12.3) |
| **Lymphoma^a^** | NA | NA | NA | NA | NA |
| **Sarcoma^a^** | NA | NA | NA | NA | NA |

^a^Lymphoma and sarcoma have been excluded from this analysis because resection is not a standard of care treatment for these cancer types.

**Table S11. Modelled changes in the average number of curative radiotherapy or chemoradiotherapy treatments without resection per year by cancer type in an initial round of multi-cancer early detection (MCED) screening and a steady-state MCED screening programme, when offered alongside current screening programmes (assuming 70% participation).** Cancer types are ordered from largest to smallest percentage change in the initial screening round.

|  |  | **Initial Screening Round** | | **Steady-State Screening Programme** | |
| --- | --- | --- | --- | --- | --- |
| **Cancer Type** | **Usual Care (n)** | **Usual Care & MCED (n)** | **Change (n [%])** | **Usual Care & MCED (n)** | **Change (%)** |
| **All Cancers Combined** | 18,534 | 23,046 | 4512 (24.3) | 19,491 | 957 (5.2) |
| **Lung** | 2146 | 4652 | 2506 (116.8) | 2945 | 799 (37.2) |
| **Pancreatic** | 52 | 100 | 48 (91.5) | 83 | 31 (59.2) |
| **Liver/Bile Duct^a^** | 8 | 15 | 7 (80.8) | 12 | 4 (41.9) |
| **Oesophageal** | 580 | 874 | 294 (50.7) | 711 | 131 (22.6) |
| **Lymphoma** | 1041 | 1483 | 442 (42.5) | 1349 | 308 (29.7) |
| **Head and Neck** | 1895 | 2511 | 616 (32.5) | 1777 | -118 (-6.2) |
| **Stomach** | 45 | 60 | 15 (31.6) | 52 | 7 (14.0) |
| **Ovarian^a^** | 1 | 2 | 1 (23.0) | 2 | 1 (45.7) |
| **Anal** | 361 | 402 | 41 (11.5) | 352 | 9 (-2.5) |
| **Prostate** | 10,178 | 10,909 | 731 (7.2) | 10,186 | 8 (0.1) |
| **Breast** | 440 | 446 | 6 (1.3) | 444 | 4 (0.7) |
| **Gallbladder^a^** | 4 | 4 | 0 (0.0) | 4 | 0 (0.0) |
| **Uterine^a^** | 53 | 53 | 0 (0.0) | 53 | 0 (0.0) |
| **Sarcoma** | 311 | 303 | 8 (-2.4) | 302 | -9 (-2.9) |
| **Kidney^a^** | 9 | 8 | 1 (-5.)8 | 9 | 0 (0.0) |
| **Cervical** | 285 | 263 | -22 (-8.0) | 257 | -28 (-9.8) |
| **Bladder** | 456 | 411 | -45 (-10.0) | 432 | -24 (-5.3) |
| **Rectal** | 456 | 364 | -92 (-20.1) | 340 | -116 (-25.4) |
| **Colon^a^** | 110 | 84 | -26 (-24) | 79 | -31 (-28.1) |

^a^Data for these cancer types have been excluded from the figures presented in the main text, because absolute change was negligible.

**Table S12. Modelled changes in the average number of tumour resections per year by cancer type in an initial round of multi-cancer early detection (MCED) screening and a steady-state MCED screening programme, when offered alongside current screening programmes (assuming 100% participation).** Cancer types are ordered from largest to smallest percentage change in the initial screening round.

|  |  | **Initial Screening Round** | | **Steady-State Screening Programme** | |
| --- | --- | --- | --- | --- | --- |
| **Cancer Type** | **Usual Care (n)** | **Usual Care & MCED (n)** | **Change (%)** | **Usual Care & MCED (n)** | **Change (%)** |
| **All Cancers Combined** | 88,915 | 129,375 | 45.5 | 101,559 | 14.2 |
| **Lung** | 5474 | 20,233 | 269.7 | 10,943 | 99.9 |
| **Pancreatic** | 715 | 1715 | 139.9 | 1220 | 70.7 |
| **Liver/Bile Duct** | 835 | 1857 | 122.4 | 1354 | 62.2 |
| **Rectal** | 4763 | 10,326 | 116.8 | 6082 | 27.7 |
| **Head and Neck** | 3820 | 7957 | 108.3 | 4772 | 24.9 |
| **Oesophageal** | 1215 | 2303 | 89.6 | 1680 | 38.3 |
| **Colon** | 11,976 | 21,406 | 78.7 | 13,703 | 14.4 |
| **Stomach** | 868 | 1474 | 69.8 | 1149 | 32.4 |
| **Anal** | 294 | 464 | 58.2 | 336 | 14.4 |
| **Cervical** | 260 | 375 | 43.8 | 394 | 51.1 |
| **Bladder** | 3112 | 4150 | 33.4 | 3389 | 8.9 |
| **Prostate** | 7232 | 7877 | 8.9 | 7248 | 0.2 |
| **Uterine** | 5358 | 5641 | 5.3 | 5449 | 1.7 |
| **Gallbladder** | 336 | 352 | 4.8 | 385 | 14.6 |
| **Breast** | 26,917 | 27,817 | 3.3 | 27,498 | 2.2 |
| **Kidney** | 4128 | 4138 | 0.2 | 4197 | 1.7 |
| **Ovarian** | 2548 | 2226 | -12.6 | 2695 | 5.8 |
| **Lymphoma^a^** | NA | NA | NA | NA | NA |
| **Sarcoma^a^** | NA | NA | NA | NA | NA |

^a^Lymphoma and sarcoma have been excluded from this analysis because resection is not a standard of care treatment for these cancer types.

**Table S13. Modelled changes in the average number of radiotherapy treatments (all intents) per year by cancer type in an initial round of multi-cancer early detection (MCED) screening and a steady-state MCED screening programme, when offered alongside current screening programmes (assuming 100% participation).** Cancer types are ordered from largest to smallest percentage change in the initial screening round.

|  |  | **Initial Screening Round** | | **Steady State Screening Programme** | |
| --- | --- | --- | --- | --- | --- |
| **Cancer type** | **Usual Care (n)** | **Usual Care & MCED (n)** | **Change (%)** | **Usual Care & MCED (n)** | **Change (%)** |
| **All cancers combined** | 60,841 | 68,720 | 13.0 | 59,147 | -2.8 |
| **Pancreatic** | 382 | 579 | 51.5 | 483 | 26.5 |
| **Lung** | 8282 | 11742 | 41.8 | 7613 | -8.1 |
| **Lymphoma** | 1814 | 2565 | 41.4 | 2335 | 28.7 |
| **Oesophageal** | 1765 | 2398 | 35.9 | 1924 | 9.0 |
| **Head and Neck** | 4831 | 6073 | 25.7 | 3915 | -19.0 |
| **Anal** | 733 | 881 | 20.2 | 713 | -2.7 |
| **Rectal** | 2707 | 2972 | 9.8 | 2147 | -20.7 |
| **Prostate** | 13,185 | 14,347 | 8.8 | 13,132 | -0.4 |
| **Stomach** | 359 | 385 | 7.4 | 348 | -3.1 |
| **Uterine** | 1997 | 2064 | 3.4 | 2016 | 1.0 |
| **Liver/Bile Duct** | 187 | 192 | 2.7 | 158 | -15.4 |
| **Breast** | 20,767 | 21,205 | 2.1 | 21,014 | 1.2 |
| **Gallbladder** | 44 | 41 | -6.8 | 45 | 0.6 |
| **Sarcoma** | 543 | 498 | -8.3 | 501 | -7.7 |
| **Cervical** | 508 | 456 | -10.3 | 449 | -11.7 |
| **Bladder** | 1090 | 939 | -13.9 | 991 | -9.1 |
| **Kidney** | 457 | 394 | -13.9 | 414 | -9.4 |
| **Ovarian^a^** | NA | NA | NA | NA | NA |
| **Colon^a^** | NA | NA | NA | NA | NA |

^a^Ovarian and colon cancer have been excluded from this analysis because radiotherapy is not a standard of care treatment for these cancer types.

**Table S14. Modelled changes in the average number of curative radiotherapy treatments per year by cancer type in an initial round of multi-cancer early detection (MCED) screening and a steady-state MCED screening programme, when offered alongside current screening programmes (assuming 100% participation).** Cancer types are ordered from largest to smallest percentage change in the initial screening round.

|  |  | **Initial Screening Round** | | **Steady-State Screening Programme** | |
| --- | --- | --- | --- | --- | --- |
| **Cancer Type** | **Usual Care (n)** | **Usual Care & MCED (n)** | **Change (%)** | **Usual Care & MCED (n)** | **Change (%)** |
| **All Cancers Combined** | 45,212 | 54,296 | 20.1 | 46,544 | 3.0 |
| **Lung** | 3010 | 7251 | 140.9 | 4239 | 40.9 |
| **Pancreatic** | 179 | 370 | 106.2 | 296 | 64.8 |
| **Liver/Bile Duct** | 20 | 37 | 87.9 | 28 | 41.4 |
| **Oesophageal** | 887 | 1511 | 70.4 | 1163 | 31.2 |
| **Lymphoma** | 1268 | 1982 | 56.3 | 1768 | 39.5 |
| **Stomach** | 90 | 129 | 43.8 | 107 | 19.4 |
| **Head and Neck** | 3956 | 5154 | 30.3 | 3287 | -16.9 |
| **Anal** | 609 | 743 | 22.2 | 601 | -1.2 |
| **Rectal** | 2116 | 2417 | 14.2 | 1730 | -18.2 |
| **Prostate** | 11,035 | 12,145 | 10.1 | 11,029 | -0.1 |
| **Gallbladder** | 11 | 12 | 4.9 | 13 | 15.4 |
| **Uterine** | 1669 | 1749 | 4.8 | 1701 | 2.0 |
| **Breast** | 18,476 | 19061 | 3.2 | 18,865 | 2.1 |
| **Sarcoma** | 367 | 348 | -5.2 | 346 | -5.7 |
| **Cervical** | 411 | 381 | -7.3 | 375 | -8.9 |
| **Kidney** | 40 | 36 | -10.1 | 37 | -6.2 |
| **Bladder** | 606 | 543 | -10.4 | 567 | -6.4 |
| **Ovarian^a^** | NA | NA | NA | NA | NA |
| **Colon^a^** | NA | NA | NA | NA | NA |

^a^Ovarian and colon cancer have been excluded from this analysis because radiotherapy is not a standard of care treatment for these cancer types.

**Table S15. Modelled changes in the average number of palliative radiotherapy treatments per year by cancer type in an initial round of multi-cancer early detection (MCED) screening and a steady-state MCED screening programme, when offered alongside current screening programmes (assuming 100% participation).** Cancer types are ordered from largest to smallest percentage decrease in the initial screening round.

|  |  | **Initial Screening Round** | | **Steady-State Screening Programme** | |
| --- | --- | --- | --- | --- | --- |
| **Cancer Type** | **Usual Care (n)** | **Usual Care & MCED (n)** | **Change (%)** | **Usual Care & MCED (n)** | **Change (%)** |
| **All Cancers Combined** | 9172 | 6676 | -27.2 | 6152 | -32.9 |
| **Head and Neck** | 309 | 153 | -50.4 | 140 | -54.6 |
| **Cervical** | 46 | 25 | -47.2 | 24 | -47.4 |
| **Breast** | 414 | 241 | -41.7 | 263 | -36.5 |
| **Rectal** | 278 | 175 | -37.2 | 150 | -46.1 |
| **Lung** | 4361 | 2852 | -34.6 | 2370 | -45.7 |
| **Anal** | 42 | 29 | -32.1 | 28 | -34.5 |
| **Uterine** | 87 | 62 | -28.4 | 70 | -19.9 |
| **Liver/Bile Duct** | 113 | 82 | -27.5 | 73 | -35.3 |
| **Bladder** | 314 | 240 | -23.3 | 265 | -15.6 |
| **Sarcoma** | 121 | 100 | -17.9 | 104 | -14.2 |
| **Kidney** | 364 | 310 | -14.9 | 327 | -10.2 |
| **Oesophageal** | 667 | 584 | -12.5 | 519 | -22.1 |
| **Gallbladder** | 28 | 25 | -10.8 | 27 | -4.2 |
| **Stomach** | 220 | 200 | -9.3 | 191 | -13.4 |
| **Lymphoma** | 342 | 313 | -8.5 | 317 | -7.3 |
| **Prostate** | 901 | 848 | -5.9 | 857 | -4.9 |
| **Pancreatic** | 158 | 150 | -5.1 | 137 | -13.4 |
| **Ovarian^a^** | NA | NA | NA | NA | NA |
| **Colon^a^** | NA | NA | NA | NA | NA |

^a^Ovarian and colon cancer have been excluded from this analysis because radiotherapy is not a standard of care treatment for these cancer types.

**Table S16. Modelled changes in the average number of cytotoxic systemic anti-cancer therapy (SACT) treatments per year by cancer type in an initial round of multi-cancer early detection (MCED) screening and a steady-state MCED screening programme, when offered alongside current screening programmes (assuming 100% participation).** Cancer types are ordered from largest to smallest percentage change in the initial screening round.

|  |  | **Initial Screening Round** | | **Steady-State Screening Programme** | |
| --- | --- | --- | --- | --- | --- |
| **Cancer Type** | **Usual Care (n)** | **Usual Care & MCED (n)** | **Change (%)** | **Usual Care & MCED (n)** | **Change (%)** |
| **All Cancers Combined** | 54,317 | 53,127 | -2.2 | 46,749 | -13.9 |
| **Pancreatic** | 2146 | 2778 | 29.5 | 2285 | 6.5 |
| **Lung** | 9069 | 11,726 | 29.3 | 7788 | -14.1 |
| **Liver/Bile Duct** | 937 | 1153 | 23.1 | 928 | -0.9 |
| **Oesophageal** | 3001 | 3586 | 19.5 | 2955 | -1.5 |
| **Anal** | 606 | 707 | 16.8 | 577 | -4.8 |
| **Bladder** | 2075 | 2258 | 8.8 | 2058 | -0.8 |
| **Stomach** | 1497 | 1621 | 8.3 | 1461 | -2.4 |
| **Breast** | 9432 | 9140 | -3.1 | 9251 | -1.9 |
| **Gallbladder** | 469 | 448 | -4.4 | 488 | 4.0 |
| **Prostate** | 2371 | 2261 | -4.6 | 2260 | -4.7 |
| **Lymphoma** | 5531 | 5070 | -8.3 | 5171 | -6.5 |
| **Kidney** | 396 | 347 | -12.5 | 363 | -8.3 |
| **Cervical** | 398 | 342 | -13.9 | 337 | -15.3 |
| **Sarcoma** | 294 | 246 | -16.3 | 254 | -13.4 |
| **Uterine** | 1023 | 825 | -19.4 | 898 | -12.2 |
| **Rectal** | 3182 | 2528 | -20.5 | 2047 | -35.7 |
| **Head and Neck** | 2219 | 1719 | -22.5 | 1264 | -43.0 |
| **Ovarian** | 2750 | 1806 | -34.3 | 2198 | -20.1 |
| **Colon** | 6458 | 4099 | -36.5 | 3702 | -42.7 |

**Table S17. Modelled changes in the average number of non-cytotoxic systemic anti-cancer therapy (SACT) treatments per year by cancer type in an initial round of multi-cancer early detection (MCED) screening and a steady-state MCED screening programme, when offered alongside current screening programmes (assuming 100% participation).** Cancer types are ordered from largest to smallest percentage change in the initial screening round.

|  |  | **Initial Screening Round** | | **Steady-State Screening Programme** | |
| --- | --- | --- | --- | --- | --- |
| **Cancer Type** | **Usual Care (n)** | **Usual Care & MCED (n)** | **Change (%)** | **Usual Care & MCED (n)** | **Change (%)** |
| **All Cancers Combined** | 4405 | 4124 | -6.4 | 3637 | -17.4 |
| **Oesophageal** | 76 | 120 | 57.7 | 93 | 22.1 |
| **Bladder** | 398 | 574 | 44.4 | 444 | 11.6 |
| **Anal** | 74 | 97 | 30.5 | 76 | 2.0 |
| **Rectal** | 226 | 276 | 22.2 | 198 | -12.4 |
| **Colon** | 52 | 60 | 14.8 | 44 | -16.2 |
| **Stomach** | 58 | 66 | 13.2 | 59 | 1.5 |
| **Liver/Bile Duct** | 135 | 149 | 10.0 | 121 | -10.7 |
| **Pancreatic** | 18 | 18 | 2.5 | 17 | -5.7 |
| **Prostate** | 271 | 277 | 2.3 | 264 | -2.6 |
| **Uterine** | 18 | 18 | 1.3 | 18 | -0.9 |
| **Gallbladder** | 1 | 1 | 0.0 | 1 | 7.5 |
| **Sarcoma** | 12 | 11 | -10.6 | 11 | -8.8 |
| **Lymphoma** | 226 | 198 | -12.6 | 203 | -10.5 |
| **Kidney** | 511 | 440 | -13.8 | 463 | -9.4 |
| **Cervical** | 38 | 33 | -14.1 | 32 | -16.6 |
| **Head and Neck** | 394 | 331 | -15.9 | 235 | -40.2 |
| **Breast** | 473 | 347 | -26.6 | 362 | -23.4 |
| **Lung** | 954 | 642 | -32.7 | 530 | -44.4 |
| **Ovarian** | 10 | 6 | -37.1 | 7 | -24.7 |

**Table S18. Modelled changes in the average number of tumour resections with or without other curative therapy (radiotherapy and/or systemic anti-cancer therapy) per year by cancer type in an initial round of multi-cancer early detection (MCED) screening and a steady-state MCED screening programme, when offered alongside current screening programmes (assuming 100% participation).** Cancer types are ordered from largest to smallest percentage change in the initial screening round.

|  |  | **Initial Screening Round** | | **Steady-State Screening Programme** | |
| --- | --- | --- | --- | --- | --- |
| **Cancer Type** | **Usual Care (n)** | **Usual Care & MCED (n)** | **Change (%)** | **Usual Care & MCED (n)** | **Change (%)** |
| **All Cancers Combined** | 77,752 | 116,418 | 49.7 | 90,630 | 16.6 |
| **Lung** | 4950 | 18,404 | 271.8 | 9946 | 101.0 |
| **Liver/Bile Duct** | 445 | 1138 | 156.0 | 812 | 82.7 |
| **Pancreatic** | 570 | 1413 | 148.1 | 1012 | 77.7 |
| **Rectal** | 4063 | 9468 | 133.0 | 5486 | 35.0 |
| **Head and Neck** | 3373 | 7536 | 123.4 | 4479 | 32.8 |
| **Colon** | 10,304 | 20,205 | 96.1 | 12,681 | 23.1 |
| **Oesophageal** | 1006 | 1945 | 93.4 | 1405 | 39.6 |
| **Stomach** | 734 | 1277 | 74.0 | 984 | 34.1 |
| **Anal** | 244 | 402 | 64.6 | 287 | 17.7 |
| **Cervical** | 236 | 346 | 46.9 | 364 | 54.5 |
| **Bladder** | 1830 | 2402 | 31.3 | 1990 | 8.8 |
| **Prostate** | 7120 | 7758 | 9.0 | 7138 | 0.3 |
| **Gallbladder** | 252 | 270 | 7.3 | 295 | 17.1 |
| **Uterine** | 4850 | 5170 | 6.6 | 4970 | 2.5 |
| **Breast** | 23,691 | 24,599 | 3.8 | 24,285 | 2.5 |
| **Kidney** | 3772 | 3820 | 1.3 | 3866 | 2.5 |
| **Ovarian** | 1799 | 1749 | -2.8 | 2115 | 17.6 |
| **Lymphoma^a^** | NA | NA | NA | NA | NA |
| **Sarcoma^a^** | NA | NA | NA | NA | NA |

^a^Lymphoma and sarcoma have been excluded from this analysis because resection is not a standard of care treatment for these cancer types.

**Table S19. Modelled changes in the average number of curative radiotherapy or chemoradiotherapy treatments without resection per year by cancer type in an initial round of multi-cancer early detection (MCED) screening and a steady-state MCED screening programme, when offered alongside current screening programmes (assuming 100% participation).** Cancer types are ordered from largest to smallest percentage change in the initial screening round.

|  |  | **Initial Screening Round** | | **Steady-State Screening Programme** | |
| --- | --- | --- | --- | --- | --- |
| **Cancer Type** | **Usual Care (n)** | **Usual Care & MCED (n)** | **Change (%)** | **Usual Care & MCED (n)** | **Change (%)** |
| **All Cancers Combined** | 18,534 | 24,979 | 34.8 | 19,902 | 7.4 |
| **Lung** | 2146 | 5726 | 166.8 | 3287 | 53.2 |
| **Pancreatic** | 52 | 121 | 130.7 | 97 | 84.6 |
| **Liver/Bile Duct** | 8 | 18 | 115.5 | 13 | 59.9 |
| **Oesophageal** | 580 | 1000 | 72.5 | 767 | 32.3 |
| **Lymphoma** | 1041 | 1672 | 60.7 | 1482 | 42.4 |
| **Head and Neck** | 1895 | 2775 | 46.5 | 1727 | -8.9 |
| **Stomach** | 45 | 66 | 45.2 | 54 | 19.9 |
| **Ovarian** | 1 | 2 | 32.9 | 2 | 65.3 |
| **Anal** | 361 | 420 | 16.5 | 348 | -3.6 |
| **Prostate** | 10,178 | 11,222 | 10.3 | 10,190 | 0.1 |
| **Breast** | 440 | 448 | 1.8 | 445 | 1.0 |
| **Gallbladder** | 4 | 4 | 1.4 | 4 | 11.3 |
| **Uterine** | 53 | 53 | 0.1 | 52 | -0.4 |
| **Sarcoma** | 311 | 300 | -3.5 | 298 | -4.2 |
| **Kidney** | 9 | 8 | -8.3 | 8 | -5.7 |
| **Cervical** | 285 | 253 | -11.4 | 245 | -14.0 |
| **Bladder** | 456 | 391 | -14.3 | 422 | -7.5 |
| **Rectal** | 456 | 325 | -28.7 | 291 | -36.3 |
| **Colon** | 110 | 72 | -34.3 | 66 | -40.2 |
